# Individual Differences in Beat Perception Affect Gait Responses to Low- and High-Groove Music

**DOI:** 10.3389/fnhum.2014.00811

**Published:** 2014-10-22

**Authors:** Li-Ann Leow, Taylor Parrott, Jessica A. Grahn

**Affiliations:** ^1^The Brain and Mind Institute, University of Western Ontario, London, ON, Canada

**Keywords:** gait rehabilitation, rhythmic auditory cueing, beat perception, basal ganglia, music rehabilitation, Parkinson’s disease, rhythmic auditory stimulation

## Abstract

Slowed gait in patients with Parkinson’s disease (PD) can be improved when patients synchronize footsteps to isochronous metronome cues, but limited retention of such improvements suggest that permanent cueing regimes are needed for long-term improvements. If so, music might make permanent cueing regimes more pleasant, improving adherence; however, music cueing requires patients to synchronize movements to the “beat,” which might be difficult for patients with PD who tend to show weak beat perception. One solution may be to use high-groove music, which has high beat salience that may facilitate synchronization, and affective properties, which may improve motivation to move. As a first step to understanding how beat perception affects gait in complex neurological disorders, we examined how beat perception ability affected gait in neurotypical adults. Synchronization performance and gait parameters were assessed as healthy young adults with strong or weak beat perception synchronized to low-groove music, high-groove music, and metronome cues. High-groove music was predicted to elicit better synchronization than low-groove music, due to its higher beat salience. Two musical tempi, or rates, were used: (1) preferred tempo: beat rate matched to preferred step rate and (2) faster tempo: beat rate adjusted to 22.5% faster than preferred step rate. For both strong and weak beat-perceivers, synchronization performance was best with metronome cues, followed by high-groove music, and worst with low-groove music. In addition, high-groove music elicited longer and faster steps than low-groove music, both at preferred tempo and at faster tempo. Low-groove music was particularly detrimental to gait in weak beat-perceivers, who showed slower and shorter steps compared to uncued walking. The findings show that individual differences in beat perception affect gait when synchronizing footsteps to music, and have implications for using music in gait rehabilitation.

## Introduction

Music and rhythm engage the motor system (Grahn and Brett, 2007; Chen et al., 2008a; Stupacher et al., 2013), ostensibly through extensive connections between the auditory and motor areas of the brain (Petrides and Pandya, 2006). The propensity of music to facilitate movement (Rossignol and Jones, 1976) has been exploited in gait rehabilitation, in which rhythmic auditory cues such as metronome tones are used to regulate movement (Rubinstein et al., 2002; Lim et al., 2005). In Parkinson’s disease (PD), a disease characterized by death of basal ganglia dopaminergic neurons (Kish, 1988), rhythmic auditory cues show promise in improving gait impairments, which are not easily treated by pharmacological interventions (Rubinstein et al., 2002; Lim et al., 2005). Patients typically show slower gait than healthy controls, mainly because they have shorter stride lengths (Morris et al., 1994a), which result from deficient internal regulation of movement amplitude and movement timing (Morris et al., 1994a). Cueing-based interventions typically require patients to synchronize footsteps to metronome cues set at either their preferred step rate or slightly faster than preferred step rate (Spaulding et al., 2012). Cueing appears to ameliorate deficient internal regulation of movement timing, not movement amplitude, by regulating step rate (Morris et al., 1994b). However, as the primary reason for slowed gait in PD is from shortened step length (smaller movement amplitude), not from slower step rate (Morris et al., 1994b), step length does not consistently increase after auditory cueing (Lim et al., 2005). Furthermore, the effect sizes of auditory cueing are not large and benefits tend not to persist over time (Nieuwboer et al., 2007). Consequently, long-term functional improvements may require that cueing become a permanent part of patients’ lives (Lim et al., 2005; Nieuwboer et al., 2007). If permanent auditory cues are required, then music, compared to metronome cues, might better motivate patients to adhere to rehabilitation regimes (de Bruin et al., 2010). Little, however, is known about exactly *what* auditory features of music, or even what task instructions, are most important to achieve the best functional outcomes in music-based therapies, limiting clinicians’ ability to optimize music for gait interventions.

Most forms of music-based movement therapy instruct patients to synchronize movements in time with the “beat,” or perceived pulse, in music. The beat is a regularly recurring perceived salience that arises in response to rhythm and music (Meyer and Cooper, 1960; London, 2012). The beat is not necessarily a strict property of a rhythmic stimulus, but rather is a psychological percept induced by the stimulus. This is why beats can be perceived through silent gaps in the music (Meyer and Cooper, 1960; London, 2012). The ability to perceive the beat differs widely across individuals (Grahn and McAuley, 2009; Grahn and Schuit, 2013; Sowinski and Dalla Bella, 2013; Launay et al., 2014). At the extreme end of the spectrum, case studies find some individuals so impaired at perceiving a beat that they are called “beat-deaf” (Phillips-Silver et al., 2011). However, less extreme impairments also exist (Grahn and McAuley, 2009). Poor beat perception is present in patients with PD as well as patients with focal basal ganglia lesions (Grahn and Brett, 2009; Schwartze et al., 2011). Not surprisingly, difficulty in perceiving the beat appears to result in difficulties in synchronizing movements to the beat in music (Phillips-Silver et al., 2011; Benoit et al., 2014). Hence, patients with PD and others with poor beat perception might benefit more from rehabilitation studies using auditory cues that have clear and unambiguous beats.

The clarity of the beat in music (i.e., beat salience) is associated with a musical characteristic called “groove” (Madison, 2006). Although groove is operationally defined as how much music evokes the desire to move (Madison, 2006), groove has also been consistently associated with greater beat salience, both when beat salience is assessed subjectively through participant ratings (Janata et al., 2012), as well as objectively through music analysis algorithms (Madison, 2006). Tapping to the beat of high-groove music is perceived to be easier than low-groove music (Madison, 2006; Janata et al., 2012). Therefore, synchronizing footsteps to the beat in high-groove music might help those with poor beat perception improve gait.

Apart from greater beat salience, high levels of groove might also improve gait by modulating an individual’s affective state. In particular, high-groove music elicits higher arousal as well as a positive affective state (Janata et al., 2012). Even at rest, high-groove music modulates excitability of the motor system more than low-groove music (Stupacher et al., 2013). Gait is sensitive to changes in the state of affect and arousal (Naugle et al., 2011). Affective properties of music can evoke faster gait (Leman et al., 2013a). This might be because altering the affective state increases movement vigor, which is a greater willingness to expend more energy for movement, such as by moving more quickly or by increasing movement amplitude (Mazzoni et al., 2007). Therefore, in addition to increasing ease of synchronization to the beat, high-groove music might also increase arousal and positive affect, such that movements during synchronization are more vigorous (i.e., faster or larger).

Overall, then, beat perception ability, beat salience, and the affective properties of music may all affect gait parameters when synchronizing movements to music. These factors might, therefore, determine the extent to which patients benefit from music-cued rehabilitation. To gain a better understanding of how beat perception ability might affect gait in complex clinical populations who often show multiple cognitive and perceptual deficits, it is important to first understand how beat perception ability affects gait in neurologically intact healthy adults. Here, we examined how beat perception ability affected gait spatiotemporal parameters when healthy adults synchronized footsteps to the beat of low-groove music, high-groove music, and metronome cues. We predicted slower and more cautious gait (i.e., slower and wider strides) with low-groove music than with high-groove music, due to the low beat salience and low arousal properties of low-groove music. To evaluate whether gait changes in the high-groove condition were primarily due to high beat salience or physiological/arousal factors, we compared the high groove and metronome conditions. Metronomes have the highest beat salience, as only the beat is present. However, metronomes have not been shown to modulate physiological arousal or affective state. Thus, if high-groove music elicits similar gait changes as metronome cues, the changes are likely due to high beat salience. If high-groove music alters gait more than metronome cues, then the affective properties of high-groove music likely also contribute to gait changes.

Beat perception ability was assessed with a perceptual beat alignment test (BAT), which measures beat perception in music with no motor requirement, and is sensitive to individual differences in beat perception ability in the general population (Iversen, 2008; Müllensiefen et al., 2012). We hypothesized that beat perception ability would affect gait spatiotemporal parameters when synchronizing footsteps to the beat in music. We divided our group into “strong” and “weak” beat-perceivers based on their BAT performance. Strong beat-perceivers were predicted to successfully maintain gait velocity while synchronizing, as beat perception would not be difficult for them. Conversely, weak beat-perceivers were predicted to show slower and more cautious gait (i.e., slower strides) while synchronizing, as beat perception would be more difficult, and could create an attention-demanding “dual task.” Finally, we predicted that the low-groove music condition would elicit slower and more cautious gait than the high-groove music condition, in both strong and weak beat-perceivers, due to its lower beat salience. We measured only the immediate effects of cueing on gait spatiotemporal parameters, not carryover effects [e.g., McIntosh et al. (1997)].

## Materials and Methods

### Participants

Forty-three healthy undergraduate psychology students from the University of Western Ontario with self-reported normal hearing (age range 18–20, 24 females) participated in this study for course credit. One participant was excluded due to incomplete data. The study was approved by the Human Research Ethics Committee at the University of Western Ontario. All participants provided written informed consent.

### Procedure

#### Beat alignment test (BAT)

We used the BAT from the Goldsmiths Music Sophistication Index v1.0 (Müllensiefen et al., 2012), which is modeled after the original BAT (Iversen, 2008). We selected the BAT because it is brief and easy to implement, and is easily used in a clinical setting. In addition, the BAT assesses beat perception in the context of music, and is therefore more directly relevant to the current step synchronization task than other assessments of beat perception, which do not use music. In the test, participants decided whether metronome beeps superimposed over instrumental music clips were correctly aligned with the perceptual beat of that clip. Beeps were aligned to the beat in four trials. The remaining trials contained either (1) a tempo error (eight trials): beeps were 2% faster or slower than the true beat tempo, or (2) a phase error (five trials): beeps were ahead of the actual beat by 10 or 17.5% of the length of the beat interval. Participants completed 3 practice trials and 17 test trials. Stimulus order was randomized for each participant. After listening to the whole clip, participants were asked to judge whether the beeps were in time with the beat by pressing the “y” key to indicate yes and the “n” key to indicate no. Participants were also asked to rate their confidence in their answer (1 = not sure, 2 = somewhat sure, 3 = very sure). Participants completed three practice trials (1 aligned, 1 tempo error, 1 phase error) to familiarize themselves with the task.

#### Step synchronization task stimulus selection

The pool of step synchronization task stimuli were originally selected based on input from lab members. Ten lab members rated a range of unfamiliar musical clips on perceived groove, and a balanced set of high and low-groove clips was selected on the basis of these ratings. Relatively obscure music was selected because strong beat-perceivers may listen to music more often, and thus be more familiar with any well-known music clips than weak beat-perceivers. Obscure music would be equally unfamiliar to all participants. As the tempo of the beat in music can be subjective (McKinney and Moelants, 2006), three lab members with musical training tapped to the beat of each music clip to determine the tempo of each song. Music clips on which raters did not all tap the same tempo were removed from the set. This resulted in a set of 20 instrumental music clips, with 10 low-groove music clips and 10 high-groove music clips. From this set, six clips were selected for each participant in the experiment, using their individual ratings (see below). For a list of stimuli and details of the selection method, see the Supplementary Material. Clip loudness was normalized to the same relative volume using Audacity (Free Software Inc., Boston, USA). Metronome sequences were created using 50 ms 1 kHz sine tones. All auditory stimuli were trimmed to start on a beat.

Prior to starting the step synchronization task, each participant rated the 20 pre-selected music clips on groove, familiarity, and enjoyment on a 10-point Likert scale, so that the final selection of clips used in the step synchronization task was tailored to each participant’s ratings. The rating scale items were as follows: (1) Groove: how much did the music make you want to move? 1 = did not want to move, 10 = very much wanted to move. (2) Familiarity: how familiar are you with the music clip? 1 = not at all familiar to me, 10 = very familiar to me. (3) How enjoyable is this piece of music? 1 = not at all enjoyable, 10 = very enjoyable. Based on individual ratings, the three music clips rated as lowest on groove and the three music clips rated as highest on groove were selected for each participant. To reduce the likelihood that familiarity with the music clip would confound the results, only low familiarity clips (ratings < 4 on familiarity) were used for the step synchronization task. Ratings data for the stimuli are listed in the Supplementary Material.

#### Step synchronization task procedure

First, each participant’s preferred gait step rate (number of steps per minute) during uncued walking was determined by having the participant walk eight lengths of a 16 foot Zeno pressure sensor walkway (one “walk” was one length of the walkway) in silence. Previous studies using similar walkways have shown that six walks results in a sufficient number of steps for reliable estimation of gait parameters (Hollman et al., 2010).The sampling rate of the walkway was 120 Hz. The tempo of each auditory stimulus (low-groove music, high-groove music, and metronome cues) was adjusted in Audacity (Free Software Inc., Boston, USA) to two tempi: (1) the participant’s preferred step rate and (2) 22.5% faster than the participant’s preferred step rate. This faster rate was selected in accordance with previous step synchronization studies in older adults (Roerdink et al., 2011). Previous work with similarly large tempo manipulations (Fujii and Schlaug, 2013) has shown that Audacity successfully preserves pitch properties of the stimuli. Auditory inspection of the stimulus waveforms did not reveal any hisses or clicks as a result of the tempo change. Participants completed 18 walking trials under the following cue conditions: low-groove music (three trials), high-groove music (three trials), and metronome sequences (three trials). Due to a technical error, for 18 participants, only 1 metronome trial was collected. One stimulus was played during each trial, and trials were completed in random order. Participants were allowed as much time as needed to find the beat before starting to walk. To reduce the effects of acceleration and deceleration on steady-state gait, walks started and finished at a line marked 1 m beyond the end of the mat, and participants were instructed to continue stepping to the beat when turning at the marked line. To prevent fatigue, participants were allowed as much time as necessary to rest between trials. Each test session lasted approximately 1 h.

### Data analysis

#### Synchronization performance

Synchronization is typically measured by assessing both phase-matching performance (the extent to which the phase of the steps matches the phase of the beats) and period-matching performance (the extent to which the tempo of the steps matches the tempo of the beats). However, we were only able to evaluate period matching performance, not phase-matching performance for the following reasons. First, it is unknown whether subjects aim to synchronize the first contact time of their step (e.g., heel-strike), the last contact time of their step (e.g., toe-offset), or some time point between the heel-strike and the toe-offset, to the beat. Synchronizing the heel-strike or the toe-offset can result in significant differences in synchronization accuracy (Chen et al., 2006). Furthermore, we do not know whether the synchronization time point within each footfall is consistent between individuals, or even between walking trials from the same individual. An estimation method that assumes that all subjects consistently synchronize at the same time point could systematically bias the data if strong beat-perceivers and weak beat-perceivers differed in their point of synchronization. Second, we could not confidently estimate beat onsets for all the music clips. Beat onsets can be irregular due to common tempo changes in music. One option is to ask musically trained individuals to tap to the music, and use those times as beat times; however, this method assumes that musically trained individuals will tap on the beat with complete accuracy and consistency throughout the full set of music clips. We attempted to objectively estimate beat onsets with beat-tracking software (BeatRoot) (Dixon, 2007), but found that BeatRoot was inaccurate in estimating the beat locations of two low-groove songs and one high-groove song. These songs were used in at least 15% of all trials across participants. Similar occasional beat-tracking inaccuracies have been noted by BeatRoot’s authors (Dixon, 2007). Finally, due to a technical problem, stimulus onset was accurately time-locked with step recordings of the pressure sensor mat for only a subset of participants: 18 weak beat-perceivers and 8 strong beat-perceivers. The small numbers of strong beat-perceivers made it unfeasible to statistically compare phase-matching performance between strong and weak beat-perceivers. We did explore phase synchronization performance using circular asynchronies across the remaining 26 datasets, assuming heel-strike as the first synchronization point, similar to previous work (McIntosh et al., 1997). These analyses yielded variable results (see Table S1 in Supplementary Material), perhaps unsurprisingly given the caveats above. We, therefore, limited our reported analyses to period-matching performance.

#### Period-matching performance

Ability to match step tempo to the stimulus tempo (i.e., period-matching accuracy) was assessed using the interbeat interval deviation (IBI deviation), which quantified how well step tempo was matched to the beat tempo on each trial (Chen et al., 2008b; Giovannelli et al., 2014). First, an automatic algorithm matched the first contact time of each step to the closest beat. Then, interstep intervals were calculated by subtracting the first contact times of consecutive steps. Interbeat intervals were calculated by subtracting beat onset times of consecutive beats. The IBI deviation was calculated by taking the absolute difference between each interstep interval and the corresponding interbeat interval. Then, to control for differences in interbeat intervals for different cue tempi, the IBI deviation was normalized to the mean interbeat interval for that trial – the absolute difference was divided by the mean interbeat interval (1).

(1) $$\text{IBI deviation}=\frac{\left|\text{mean interstep interval}-\text{interbeat interval}\right|}{\text{mean interbeat interval}}$$

Variability of period matching was assessed using the standard deviation of the IBI deviation.

#### Spatiotemporal gait parameters

Based on previous gait studies (Hollman et al., 2011), six gait parameters of interest were selected for analysis: stride velocity, step length, step time, double support time, stride width, and step length coefficient of variability. Gait speed was determined from stride velocity [the distance covered per unit time (cm/s) for every two consecutive steps]. Step length was the anterior–posterior distance from the first contact location of one step to the first contact location of the next step. Step time was the interval between the first contact time of one footprint to the first contact time of the next footprint. Thus, changes in velocity could result from changes in step length and/or changes in step time. In addition, to assess the attentional demands of gait synchronization, we also assessed double support time (% of time that both feet were simultaneously in contact with the ground), stride width distance between a line connecting two ipsilateral foot heel contacts (the stride) and the contralateral foot heel contact between those events, measured perpendicular to the stride, and step length coefficient of variation (standard deviation of step length normalized to the mean step length) (Hollman et al., 2011). Longer double support time, wider strides, and greater step length variability are associated with more cautious gait as a result of greater attentional demand during gait (Al-Yahya et al., 2011).

#### Statistical analyses

We were interested in how gait changed during the different music and metronome conditions compared to uncued walking. Hence, we obtained change scores of each gait parameter by subtracting the average gait parameters in each stimulus condition from the average gait parameters in uncued walking (Rochester et al., 2005). Then, to enable comparisons across individuals to be made on the same scale (e.g., a long-legged participant who takes long steps may have a greater absolute difference in step length than a short-legged participant who takes short steps), we normalized these change scores to gait parameters obtained from uncued walking.

(2) $$\begin{array}{l} \text{Normalized change score}=\frac{\text{Gait parameter}-\text{Uncued gait parameter}}{\text{Uncued gait parameter}} \end{array}$$

To evaluate how beat perception ability affected gait synchronization to low-groove music, high-groove music, and metronome, Beat Perception (weak, strong beat-perceivers) by Cue (low groove, high groove, metronome) by Tempo (preferred step rate, faster step rate) mixed-measures ANOVAs were conducted for each gait parameter of interest. The Greenhouse-Geisser correction was applied when Mauchly’s test of sphericity was significant. Pairwise comparisons using Dunn-Sidak corrections for multiple comparisons identified significant differences between conditions.

## Results

### Beat alignment test scores

Beat alignment test scores were calculated as the proportion correct from all 17 trials. Scores ranged from 0.47 to 1, *M* = 0.69, SD = 0.15, consistent with previous findings (Iversen, 2008; Grahn and Schuit, 2013). A split of 0.65 was used to classify participants as strong beat-perceivers (scores above 0.65, *n* = 19) and weak beat-perceivers (scores equal to or below 0.65, *n* = 23). The use of the 0.65 split was justified by similar BAT median scores in other versions of the BAT (Iversen, 2008; Grahn and Schuit, 2013). Strong beat-perceivers had more years of musical training (*M* = 5.11, SD = 4.45, SEM = 1.05) than weak beat-perceivers (*M* = 2.91, SD = 3.51, SEM = 0.74), although this difference was not statistically significant [*t*(39) = 1.77, p = 0.08].

### Period-matching performance

**Figure 1:**
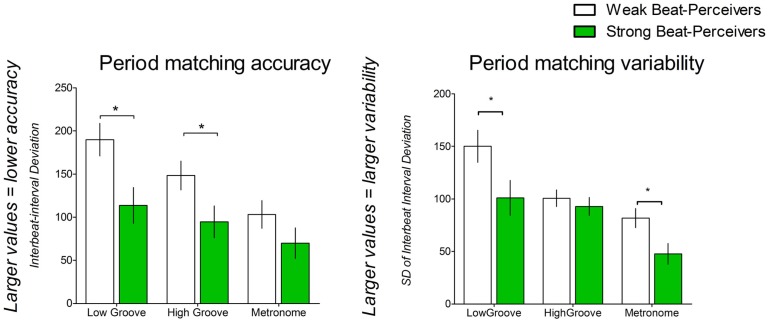
**Period-matching accuracy as estimated by IBI deviation, and period-matching variability as estimated by standard deviation of the IBI deviation**. Weak beat-perceivers are shown in clear bars; strong beat-perceivers are shown in green bars.

Figure 1, top panel, shows period-matching accuracy, or how well participants matched step tempo to cue tempo, as indicated by interbeat interval deviation. Smaller values indicate more accurate period matching. Only the faster tempo condition was analyzed, as the cues at preferred tempo were specifically matched to each participant’s preferred step tempo; therefore, the periods were expected to match regardless of synchronization ability. Weak beat-perceivers were worse at matching step tempo to the cue tempo than strong beat-perceivers (see Figure 1), as shown by a significant main effect of Beat Perception [*F*(1,40) = 5.92, *p* = 0.02, η^2^_p_ = 0.13]. A significant main effect of Cue [*F*(2,80) = 17.46, *p* < 0.001, η^2^_p_ = 0.30] indicates that period-matching accuracy differed between cue types. Period matching was best for metronome cues (86.53 ± 12.06), followed by high-groove music (121.44 ± 12.43), and was worst for low-groove music (151.79 ± 14.04). All conditions differed significantly from each other. Period matching was significantly less accurate for low-groove music compared to high-groove music (*p* = 0.001), and for low-groove music compared to metronome cues (*p* < 0.001). Period matching was also significantly less accurate for high-groove music compared to metronome cues (*p* = 0.003). The Cue × Beat Perception interaction was not significant: [*F*(2,80) = 1.89, *p* = 0.16, η^2^_p_ = 0.04], indicating that period matching in strong and weak beat-perceivers was similarly affected by the different cue types.

The variability of period matching was indicated by the standard deviation of the IBI deviation (see Figure 1), with smaller values indicating less variable period matching. Weak beat-perceivers showed more variable period matching for all cues than strong beat-perceivers, as shown by a significant main effect of Beat Perception [*F*(1,40) = 5.79, *p* = 0.02, η^2^_p_ = 0.13]. A significant main effect of Cue [*F*(2,80) = 20.88, *p* < 0.001, η^2^_p_ = 0.34] indicated that variability differed between cue types. Period matching was least variable for metronome cues (64.73 ± 6.71), followed by high-groove music (96.68 ± 5.87), and was most variable for low-groove music (125.50 ± 11.33). All conditions differed significantly from each other. Period matching was more variable for low-groove music compared to high-groove music (*p* = 0.036), and more variable for low-groove music compared to metronome cues (*p* < 0.001). Period matching was more variable for high-groove music than for metronome cues (*p* < 0.001). The Cue × Beat Perception interaction was not significant [*F*(2,80) = 2.45, *p* = 0.09, η^2^_p_ = 0.058].

### Gait parameters for different cue conditions

Descriptive statistics for gait parameters measured in each cue condition are shown in Table 1.

**Table 1:** **Means and standard deviations of gait parameters for each cueing condition when cue tempo was set at preferred step tempo and at 22.50% faster than preferred tempo, averaged across all participants**.

	Preferred tempo	Faster tempo (22.50% faster)
**STRIDE VELOCITY (CM/S)**		
Uncued	95.4 (14.4)	N/A
Low groove	88.4 (16.8)*	93.3 (22.0)
High groove	95.5 (18.6)	107.7 (20.9)*
Metronome	95.2 (17.3)	103.8 (19.2)*
**STEP LENGTH (CM)**		
Uncued	61.9 (6.5)	N/A
Low groove	61.0 (7.4)*	61.2 (8.2)*
High groove	59.8 (7.4)	58.8 (8.0)*
Metronome	60.8 (7.0)	59.9 (7.7)
**STEP TIME (S)**		
Uncued	0.37 (0.17)	N/A
Low groove	0.41 (0.18)*	0.29 (0.24)*
High groove	0.37 (0.17)	0.34 (0.16)*
Metronome	0.37 (0.17)	0.22 (0.25)*
**STRIDE WIDTH (CM)**		
Uncued	7.0 (2.5)	N/A
Low groove	8.6 (2.9)*	8.5 (2.7)*
High groove	7.8 (2.9)*	8.0 (2.9)*
Metronome	8.1 (2.7)*	7.2 (2.4)
**DOUBLE SUPPORT (%)**		
Uncued	25.62 (2.82)	N/A
Low groove	25.55 (3.04)	25.22 (3.18)
High groove	25.26 (3.23)	24.92 (3.49)
Metronome	24.97 (3.13)*	24.23 (3.33)*
**STRIDE LENGTH VARIABILITY (CV)**		
Uncued	0.051 (0.021)	N/A
Low groove	0.068 (0.022)*	0.070 (0.027)*
High groove	0.062 (0.018)*	0.072 (0.026)*
Metronome	0.055 (0.019)	0.057 (0.015)

Asterisks (*) indicate that the gait parameter for the cueing condition differed significantly (*p* < 0.05) from uncued gait.

#### Stride velocity, step length, and step time

**Figure 2:**
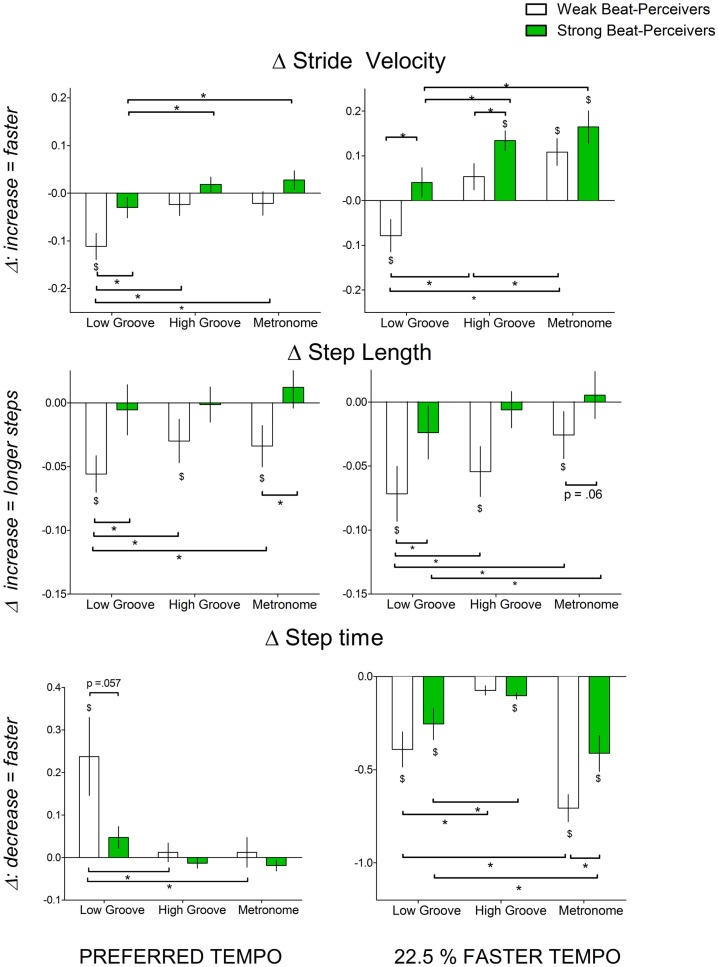
**Normalized change scores in gait parameters indicating gait speed (stride velocity (cm/s), step length (cm), step time (s)) with all three cue types (low-groove music, high-groove music, metronome cues) at different tempi: left panel, preferred tempo and right panel, faster tempo**. All normalized change scores shown are a proportion of uncued gait. Weak beat-perceivers are shown in clear bars; strong beat-perceivers are shown in green bars. Values close to zero show that gait parameters showed little change compared to uncued walking. Dollar signs ($) indicate significant differences relative to uncued walking at *p* < 0.05. Asterisks (*) indicate significance differences at *p* < 0.05.

Figure 2 shows the normalized change scores for stride velocity, step length, and step time. Left panels of Figure 2 show that at preferred tempo, weak beat-perceivers showed slower stride velocity than strong beat-perceivers across all three cue types. This was large because weak beat-perceivers (clear bars) tended to reduce step length during cueing, unlike strong beat-perceivers (green bars), who successfully maintained step length. Right panels of Figure 2 show that at the faster tempo, weak beat-perceivers (clear bars) sped up stride velocity while shortening step length and step time (right panel, middle graph), indicating that weak beat-perceivers sped up stride velocity by taking faster but shorter steps, whereas strong beat-perceivers sped up stride velocity by taking faster steps of similar length to those during uncued walking. The main effects of Beat Perception were significant for stride velocity [*F*(1, 40) = 5.15, *p* = 0.029, η^2^_p_ = 0.11] and near significant for step length [*F*(1, 40) = 3.21, *p* = 0.08, η^2^_p_ = 0.07]. The main effects of Beat Perception were not qualified by any significant interactions with Cue or Tempo.

There were significant Cue × Tempo interactions for stride velocity [*F*(2, 80) = 11.027, *p* < 0.001, η^2^_p_ = 0.22] and step length [*F*(2, 80) = 3.21, *p* = 0.04, η^2^_p_ = 0.07]. This interaction was because high-groove music elicited similar stride velocity and step length to metronome cues at preferred tempo [stride velocity: *t*(41) = 0.5, *p* = 0.62, step length: *t*(41) = 0.53, *p* = 0.60], but elicited slower and shorter steps than metronome cues at the faster tempo [stride velocity: *t*(41) = 2.99, *p* = 0.005, step length: [*t*(41) = 2.96, *p* = 0.005]. Low-groove music elicited significantly slower and shorter steps than high-groove music and metronome (see Figure 2), both at preferred tempo [stride velocity: *t*(41) = 4.62, *p* < 0.001, step length *t*(41) = 2.43, *p* = 0.019] and at faster tempo [*t*(41) = 5.60, *p* < 0.001, step length *t*(41) = 2.14, *p* = 0.039].

For step time, there was a significant Beat Perception × Cue × Tempo interaction [*F*(1,40) = 7.52, *p* < 0.001, η^2^_p_ = 0.16]. This three-way interaction was because, at preferred tempo, weak beat-perceivers slowed step times more than strong beat-perceivers with low-groove music [*t*(25.29) = 1.99, *p* = 0.057] but not with high-groove music [*t*(40) = 0.97, *p* = 0.34] and metronome cues [*t*(40) = 0.77, *p* = 0.45]. At the faster tempo, weak beat-perceivers sped up step times more than strong beat-perceivers with metronome cues [*t*(40) = 2.47, *p* = 0.018] but not with low-groove music [*t*(40) = 1.07, *p* = 0.29] or high-groove music [*t*(40) = 0.82, *p* = 0.42]. With faster tempo metronome cues, the markedly briefer step times in weak beat-perceivers were accompanied by reduced step length, and thus, the increase in stride velocity to fast metronome cues (Figure 2, top right panel, clear bars) was accomplished by taking shorter but faster steps.

#### Stride width, double support time, and stride length variability

**Figure 3:**
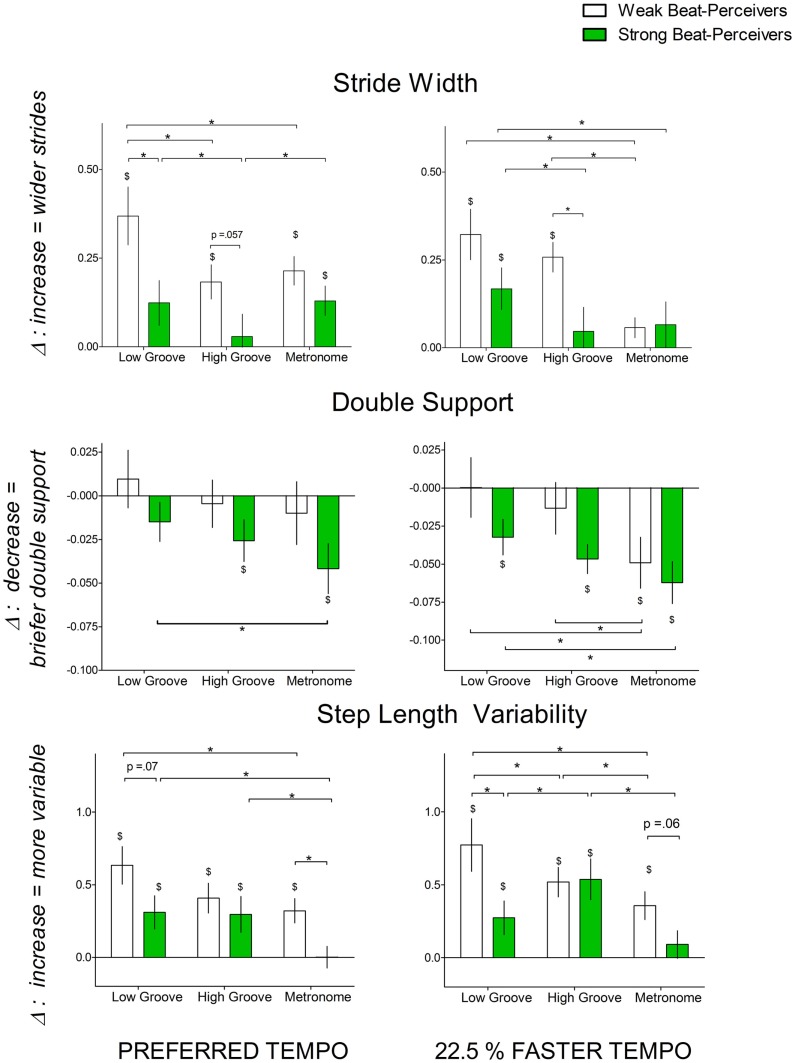
**Normalized change scores for gait parameters indicating the attentional demands of synchronization [stride width (cm), double support time (% of gait cycle), and step length variability] with each cue type (low-groove music, high-groove music, metronome cues) at different tempi: left panel, preferred tempo and right panel, faster tempo**. All normalized change scores here are a proportion of uncued gait. Weak beat-perceivers are shown in clear bars; strong beat-perceivers are shown in green bars. Values close to zero show that gait parameters showed little change compared to uncued walking. Dollar signs ($) indicate significant differences relative to uncued walking at *p* < 0.05. Asterisks (*) indicate significance differences between conditions at *p* < 0.05.

Wider strides, longer double support time, and greater stride length variability indicate greater attentional demands and greater cautiousness during gait (Al-Yahya et al., 2011). Figure 3 shows the normalized change scores for stride width, stride length variability, and double support time. For stride width, significant main effects of Beat Perception [*F*(1,40) = 4.41, *p* = 0.04, η^2^_p_ = 0.099] and Cue [*F*(2,80) = 9.64, *p* < 0.001, η^2^_p_ = 0.19] were qualified by a significant three-way Beat Perception × Cue × Tempo interaction [*F*(1,40) = 3.27, *p* = 0.043, η^2^_p_ = 0.076]. At preferred tempo, weak beat-perceivers increased stride width more than strong beat-perceivers with low-groove music [*t*(40) = 2.29, *p* = 0.027], but not with high-groove music [*t*(40) = 1.96, *p* = 0.056], or with metronome cues [*t*(40) = 1.45, *p* = 0.15]. At the faster tempo, weak beat-perceivers increased stride width more than strong beat-perceivers with high-groove music [*t*(40) = 2.71, *p* = 0.01], but not with low-groove music [*t*(40) = 1.61, *p* = 0.12], or with metronome cues [*t*(40) = 0.13, *p* = 0.9].

For double support time, the main effect of Beat Perception was not significant [*F*(1,40) = 2.05, *p* = 0.16, η^2^_p_ = 0.05], and there were no significant interactions between Beat Perception and other factors. There was a significant main effect of Cue [*F*(2,40) = 7.85, *p* = 0.001, η^2^_p_ = 0.16], which was not qualified by any interactions, as both strong and weak beat-perceivers decreased double support time more with metronome cues than with low-groove music (*p* = 0.001) or high-groove music (*p* = 0.049).

For stride length variability, the marginally significant main effect of Beat Perception [*F*(1,40) = 3.46, *p* = 0.07, η^2^_p_ = 0.08] was qualified by a significant Cue x Beat Perception interaction [*F*(2,40) = 4.40, *p* = 0.015, η^2^_p_ = 0.10], as weak beat-perceivers showed greater increases in stride length variability than strong beat-perceivers with low-groove music [preferred: *t*(40) = 1.84, *p* = 0.07, faster: *t*(40) = 2.22, *p* = 0.03], and metronome cues [preferred: *t*(40) = 2.79, *p* = 0.008, faster: *t*(40) = 1.96, *p* = 0.05], but not with high-groove music [preferred: *t*(40) = 0.71, *p* = 0.48, faster: *t*(40) = 0.11, *p* = 0.91].

In summary, weak beat-perceivers showed larger increases in stride width and stride length variability than strong beat-perceivers, and this pattern of results was particularly evident with low-groove music.

## Discussion

The primary finding of this study is that individual differences in beat perception ability determined whether gait was maintained or impaired by synchronizing to music and metronome cues. Weak beat-perceivers showed slower gait than strong beat-perceivers, perhaps because weak beat-perceivers had more difficulty in synchronizing footsteps to the beat, as shown by poorer performance in period matching their step tempo to the cue tempo. Conversely, strong beat-perceivers showed faster gait than weak beat-perceivers, perhaps because strong beat-perceivers found it easier to synchronize footsteps to the beat, as shown by better period-matching performance in strong beat-perceivers than weak beat-perceivers. Collectively, these findings suggest that beat perception ability may affect outcomes in music-based gait rehabilitation, particularly in patients with PD, who show beat perception deficits (Grahn and Brett, 2009), as well as cautious, less vigorous movement (Mazzoni et al., 2007).

### Gait responses to auditory cues depend on beat perception ability

Weak beat-perceivers showed overall slower, shorter, and wider steps than strong beat-perceivers when synchronizing to auditory cues, regardless of cue tempo. These effects were particularly evident with low-groove music, which reduced step length at both preferred and faster tempos in weak beat-perceivers. Slower, shorter and wider strides are commonly evoked by greater attentional demands in dual task paradigms [for a review, see Al-Yahya et al. (2009)]. Therefore, the shorter, slower, and wider steps in weak beat-perceivers might be because synchronizing movements to auditory cues is more attention-demanding for weak beat-perceivers than strong beat-perceivers. Our findings suggest that for weak beat-perceivers, synchronizing footsteps to the beat might be an attention-demanding task that slows and shortens strides.

In weak beat-perceivers, negative effects of synchronization on gait parameters (e.g., step length and double support time) were evident even with metronome cues, when there was no need to extract the beat structure. Such findings appear at first glance to contradict previous studies that report that weak beat-perceivers show intact synchronization performance when tapping to metronome cues (Sowinski and Dalla Bella, 2013; Launay et al., 2014). However, as only the timing of finger tapping was assessed in these previous studies, it remains possible that weak beat-perceivers might alter spatial kinematics when tapping to metronome cues similarly to the gait alterations observed here. Walking might also be more sensitive than finger tapping to individual differences in beat perception ability – recent work has shown that reproducing a rhythm by walking is more difficult than by finger tapping or foot tapping (Iannarilli et al., 2013). Thus, gait synchronization may be more likely to be affected by poorer beat perception than finger tapping synchronization.

### Effect of cue properties on synchronization accuracy and gait parameters

Low-groove music appears to be harder to synchronize to, and also to have a generally detrimental effect on gait. Tempo-matching was less accurate and more variable with low-groove music compared to high-groove music and compared to metronome, as low-groove music elicited larger and more variable deviations from interbeat intervals. This difficulty cannot be explained by differences in tempo, as the same tempo manipulations were done for low-groove music and high-groove music. Low-groove music was particularly detrimental to gait kinematics, as it elicited slower, shorter, and wider steps compared to uncued walking. High-groove music and metronome cues elicited similar effects on relevant gait parameters such as stride velocity and step length, suggesting that high-groove music might be a viable alternative to metronome cues.

Our finding that high-groove music did not elicit faster, longer steps than metronome cues appears inconsistent with previous findings of faster stride velocity with music than with metronome cues (Styns et al., 2007; Leman et al., 2013b). Several methodological differences might explain the difference in results. First, highly familiar music was used in these previous studies (Styns et al., 2007), unlike the current study, which used unfamiliar music. Familiarity with the beat structure of the music might increase the ease of extracting the beat and synchronization to the beat, therefore, reducing the attentional demands of synchronization that could counteract positive effects of groove on step length. Studies that directly compare the effects of low and high familiarity music would be needed to determine whether familiarity is important for high-groove music to elicit more beneficial effects than metronome cues. Another approach may be to accent the beat structure of low familiarity music with metronome cues. This would reduce the attentional demands of beat extraction, potentially improving gait outcomes, especially for weak beat-perceivers.

### Auditory cues increased gait velocity by altering step time, not step length

Under all cueing conditions, even when cue tempo was sped up, step length did not significantly increase compared to uncued walking. Stride velocity increased, but by decreasing step time, not by increasing step length. That is, participants moved faster, but by taking briefer and more frequent steps, not by taking longer steps. These findings are consistent with previous findings of shorter (Cubo et al., 2004; Dibble et al., 2004) or unaltered step lengths during synchronization to metronome cues, both for patients with PD (Morris et al., 1994a; Howe et al., 2003; Almeida et al., 2007) and healthy adults (Wittwer et al., 2013). These findings are important because the slowing of gait in PD results primarily from steps that are too short (Morris et al., 1994b, 1996), thus simply presenting faster cues may not help increase step length (Morris et al., 1994a). In fact, several studies suggest that metronome cueing only lengthens steps if patients with PD are also intentionally increasing step lengths while stepping in time to the cue (Nieuwboer et al., 2007; Baker et al., 2008; Rochester et al., 2011). Therefore, synchronizing footsteps to auditory cues might not be sufficient to elicit increased step length: intention to increase step length might also be necessary.

### Implications for gait rehabilitation in Parkinson’s disease

In the current study, gait performance depended strongly on beat perception ability. The effects of beat perception ability might explain previous reports of variable outcomes of music-based rehabilitation in PD [for a review, see de Dreu et al. (2012)]. Functional neuroimaging studies demonstrate involvement of the basal ganglia in internally generating and maintaining the beat (Grahn and Rowe, 2013). Deficient basal ganglia function impairs beat perception in patients with PD (Grahn and Brett, 2009) and patients with focal basal ganglia lesions (Schwartze et al., 2011), suggesting that intact basal ganglia function might be necessary to perceive the beat. Difficulty generating and maintaining the beat could limit the ability of patients with PD to improve from rehabilitation paradigms when they are required to synchronize footsteps to the beat (Nombela et al., 2013). Weaker beat perception in patients with PD might further increase the attentional demand of synchronizing footsteps to the beat, thereby worsening gait in patients with PD, as they generally show weaker attentional control than healthy individuals when walking (Yogev et al., 2005).

For patients with PD with weak beat perception, synchronization to the beat in music might elicit weaker gait performance than synchronization to metronome cues. One way of reducing the difficulty of synchronization in weak beat-perceivers is to make the musical beat structure unambiguous by embedding metronome cues into music. This method of embedding music with metronome cues has previously elicited faster and longer strides in comparison to silence (Thaut et al., 1996; McIntosh et al., 1997) and in comparison to metronome cues alone (Wittwer et al., 2013). Music combined with metronome cues might therefore evoke additive effects that facilitate longer, faster strides. In addition, using familiar music might be beneficial, as familiarity with music and consequently familiarity with the beat structure might reduce the attentional demands of extracting the beat, such that gait might be less impaired in weak beat-perceivers. This is consistent with the finding that patients with PD increased stride velocity and stride length when they synchronized footsteps to the beat of highly familiar music for 30 min daily over 3 weeks (de Bruin et al., 2010).

### Limitations

The BAT perception test is a brief measure of beat perception ability, but does not provide as much information as more extensive beat perception measures, such as the BAASTA (Benoit et al., 2014) or the Harvard BAT (Fujii and Schlaug, 2013). We also did not test for pitch perception deficits, which have previously been shown to result in difficulties with rhythm perception (Foxton et al., 2006). The BAT was selected because it uses ecologically valid music, similar to that used during walking, and it is brief, easily implemented, and also practical to administer in rehabilitation contexts. Even with these limitations, the categorization of strong and weak beat-perceivers revealed distinct gait responses when synchronizing to the beat: participants classified as weak beat-perceivers by this task showed overall worse gait performance when required to synchronize footsteps to the beat. The BAT perception test might, therefore, be useful in tailoring gait rehabilitation protocols for weak and strong beat-perceivers. While weak beat-perceivers might benefit from increasing the beat salience of music by embedding metronome cues into music, strong beat-perceivers might not require this, and embedding metronomes may even reduce their enjoyment of the rehabilitation. It remains to be seen if the BAT is sensitive enough to characterize beat perception ability in more variable populations such as older adults or neurologically impaired patients such as individuals with PD. In addition, the current study did not examine short-term carryover effects of cueing on gait, which have been reported for clinical populations such as PD (Thaut et al., 1996). Future examinations could determine which stimulus cue properties provide the longest persistence of carryover benefits in clinical populations.

Another limitation of this study, and our understanding of gait synchronization more generally, is that it is unclear which timepoint within each footfall is synchronized to the beat. In bipedal gait, each foot stays in contact with the ground for long periods of time (up to 800 ms in our data), and thus foot contact times can be longer than the interbeat interval. This contrasts with assessments of synchronization performance with finger tapping, in which the finger contact time is brief and discrete. In bipedal gait, participants could synchronize any timepoint between the first contact time (typically the heel-strike) and the last contact time of the step (the toe-off). Synchronizing using the heel-strike or the toe-off can result in significant differences in synchronization accuracy (Chen et al., 2006). Although previous work estimated synchronization performance using the first contact time (Thaut et al., 1996), there is no clear evidence that individuals intend for that to be the synchronized point. Furthermore, we do not know whether the synchronized time point is consistent between individuals, conditions, or even within each walking trial. To assess synchronization accuracy, future studies will need to determine what timepoint of the footfall is synchronized, and whether strong and weak beat-perceivers differ in their selection of the synchronization timepoint.

## Summary

The primary finding of this study is that beat perception ability affects whether gait performance is impaired or maintained when synchronizing footsteps to the beat in music. When synchronizing to auditory cues, strong beat-perceivers either maintained or improved gait performance, whereas weak beat-perceivers showed slower, more cautious gait, particularly with low-groove music, in which beat locations are less salient. High-groove music and metronome cues generally resulted in better gait performance than low-groove music in both weak and strong beat-perceivers. Taken together, these findings suggest that tailoring auditory cues according to patient beat perception ability in gait rehabilitation might elicit better outcomes.

## Conflict of Interest Statement

The authors declare that the research was conducted in the absence of any commercial or financial relationships that could be construed as a potential conflict of interest.

## Supplementary Material

The Supplementary Material for this article can be found online at http://www.frontiersin.org/Journal/10.3389/fnhum.2014.00811/abstract

## References

[B1] Almeida Q. J. Frank J. S. Roy E. A. Patla A. E. Jog M. S. (2007). Dopaminergic modulation of timing control and variability in the gait of Parkinson’s disease. Mov. Disord. 22, 1735–1742. 10.1002/mds.21603 17557356

[B2] Al-Yahya E. Dawes H. Collett J. Howells K. Izadi H. Wade D. T. (2009). Gait adaptations to simultaneous cognitive and mechanical constraints. Exp. Brain Res. 199, 39–48. 10.1007/s00221-009-1968-1 19672583

[B3] Al-Yahya E. Dawes H. Smith L. Dennis A. Howells K. Cockburn J. (2011). Cognitive motor interference while walking: a systematic review and meta-analysis. Neurosci. Biobehav. Rev. 35, 715–728. 10.1016/j.neubiorev.2010.08.008 20833198

[B4] Baker K. Rochester L. Nieuwboer A. (2008). The effect of cues on gait variability – reducing the attentional cost of walking in people with Parkinson’s disease. Parkinsonism Relat. Disord. 14, 314–320. 10.1016/j.parkreldis.2007.09.008 17988925

[B5] Benoit C. E. Dalla Bella S. Farrugia N. Obrig H. Mainka S. Kotz S. A. (2014). Musically cued gait-training improves both perceptual and motor timing in Parkinson’s disease. Front. Hum. Neurosci. 8, 494. 10.3389/fnhum.2014.00494 25071522 PMC4083221

[B6] Chen H. Y. Wing A. M. Pratt D. (2006). The synchronisation of lower limb responses with a variable metronome: the effect of biomechanical constraints on timing. Gait Posture 23, 307–314. 10.1016/j.gaitpost.2005.04.001 15894483

[B7] Chen J. L. Penhune V. B. Zatorre R. J. (2008a). Listening to musical rhythms recruits motor regions of the brain. Cereb. Cortex 18, 2844–2854. 10.1093/cercor/bhn042 18388350

[B8] Chen J. L. Penhune V. B. Zatorre R. J. (2008b). Moving on time: brain network for auditory-motor synchronization is modulated by rhythm complexity and musical training. J. Cogn. Neurosci. 20, 226–239. 10.1162/jocn.2008.20018 18275331

[B9] Cubo E. Leurgans S. Goetz C. G. (2004). Short-term and practice effects of metronome pacing in Parkinson’s disease patients with gait freezing while in the ‘on’ state: randomized single blind evaluation. Parkinsonism Relat. Disord. 10, 507–510. 10.1016/j.parkreldis.2004.05.001 15542012

[B10] de Bruin N. Doan J. B. Turnbull G. Suchowersky O. Bonfield S. Hu B. (2010). Walking with music is a safe and viable tool for gait training in Parkinson’s disease: the effect of a 13-week feasibility study on single and dual task walking. Parkinsons Dis. 2010, 483530. 10.4061/2010/483530 20976086 PMC2957229

[B11] de Dreu M. J. Van Der Wilk A. S. D. Poppe E. Kwakkel G. Van Wegen E. E. H. (2012). Rehabilitation, exercise therapy and music in patients with Parkinson’s disease: a meta-analysis of the effects of music-based movement therapy on walking ability, balance and quality of life. Parkinsonism Relat. Disord. 18(Suppl. 1), S114–S119. 10.1016/S1353-8020(11)70036-0 22166406

[B12] Dibble L. E. Nicholson D. E. Shultz B. Macwilliams B. A. Marcus R. L. Moncur C. (2004). Sensory cueing effects on maximal speed gait initiation in persons with Parkinson’s disease and healthy elders. Gait Posture 19, 215–225. 10.1016/S0966-6362(03)00065-1 15125910

[B13] Dixon S. (2007). Evaluation of the audio beat tracking system beat root. J. New Music Res. 36, 39–50. 10.1080/09298210701653310

[B14] Foxton J. M. Nandy R. K. Griffiths T. D. (2006). Rhythm deficits in ‘tone deafness’. Brain Cogn. 62, 24–29. 10.1016/j.bandc.2006.03.005 16684584

[B15] Fujii S. Schlaug G. (2013). The Harvard Beat Assessment Test (H-BAT): a battery for assessing beat perception and production and their dissociation. Front. Hum. Neurosci. 7:771. 10.3389/fnhum.2013.00771 24324421 PMC3840802

[B16] Giovannelli F. Innocenti I. Rossi S. Borgheresi A. Ragazzoni A. Zaccara G. (2014). Role of the dorsal premotor cortex in rhythmic auditory-motor entrainment: a perturbational approach by rTMS. Cereb. Cortex. 24, 1009–1016. 10.1093/cercor/bhs386 23236203

[B17] Grahn J. A. Brett M. (2007). Rhythm and beat perception in motor areas of the brain. J. Cogn. Neurosci. 19, 893–906. 10.1162/jocn.2007.19.5.893 17488212

[B18] Grahn J. A. Brett M. (2009). Impairment of beat-based rhythm discrimination in Parkinson’s disease. Cortex 45, 54–61. 10.1016/j.cortex.2008.01.005 19027895

[B19] Grahn J. A. McAuley J. D. (2009). Neural bases of individual differences in beat perception. Neuroimage 47, 1894–1903. 10.1016/j.neuroimage.2009.04.039 19376241

[B20] Grahn J. A. Rowe J. B. (2013). Finding and feeling the musical beat: striatal dissociations between detection and prediction of regularity. Cereb. Cortex 23, 913–921. 10.1093/cercor/bhs083 22499797 PMC3593578

[B21] Grahn J. A. Schuit D. (2013). Individual differences in rhythmic ability: Behavioral and neuroimaging investigations. Psychomusicology 22, 105–121. 10.1037/a0031188

[B22] Hollman J. H. Childs K. B. Mcneil M. L. Mueller A. C. Quilter C. M. Youdas J. W. (2010). Number of strides required for reliable measurements of pace, rhythm and variability parameters of gait during normal and dual task walking in older individuals. Gait Posture 32, 23–28. 10.1016/j.gaitpost.2010.02.017 20363136

[B23] Hollman J. H. Mcdade E. M. Petersen R. C. (2011). Normative spatiotemporal gait parameters in older adults. Gait Posture 34, 111–118. 10.1016/j.gaitpost.2011.03.024 21531139 PMC3104090

[B24] Howe T. Lovgreen B. Cody F. Ashton V. Oldham J. (2003). Auditory cues can modify the gait of persons with early-stage Parkinson’s disease: a method for enhancing parkinsonian walking performance? Clin. Rehabil. 17, 363–367. 10.1191/0269215503cr621oa 12785243

[B25] Iannarilli F. Vannozzi G. Iosa M. Pesce C. Capranica L. (2013). Effects of task complexity on rhythmic reproduction performance in adults. Hum. Mov. Sci. 32, 203–213. 10.1016/j.humov.2012.12.004 23452943

[B26] Iversen J. R. Patel A. D. (2008). “The beat alignment test (BAT): surveying beat processing abilities in the general population,” in Proceedings of the 10th International Conference on Music Perception and Cognition (ICMPC10). eds Miyazaki K. Hiraga Y. Adachi M. Nakajima Y. Tsuzaki M. Sapporo 465–468

[B27] Janata P. Tomic S. T. Haberman J. M. (2012). Sensorimotor coupling in music and the psychology of the groove. J Exp Psychol 141, 54–75. 10.1037/a0024208 21767048

[B28] Kish S. J. (1988). Uneven pattern of dopamine loss in the striatum of patients with idiopathic Parkinson’s disease. N. Engl. J. Med. 318, 876–880. 10.1056/NEJM198804073181402 3352672

[B29] Launay J. Grube M. Stewart L. (2014). Dysrhythmia: a specific congenital rhythm perception deficit. Front. Psychol. 5:18. 10.3389/fpsyg.2014.00018 24550854 PMC3913998

[B30] Leman M. Moelants D. Varewyck M. Styns F. Van Noorden L. Martens J.-P. (2013a). Activating and relaxing music entrains the speed of beat synchronized walking. PLoS ONE 8:e67932–e67932. 10.1371/journal.pone.0067932 23874469 PMC3707869

[B31] Leman M. Moelants D. Varewyck M. Styns F. Van Noorden L. Martens J. P. (2013b). Activating and relaxing music entrains the speed of beat synchronized walking. PLoS ONE 8:e67932. 10.1371/journal.pone.0067932 23874469 PMC3707869

[B32] Lim I. Van Wegen E. De Goede C. Deutekom M. Nieuwboer A. Willems A. (2005). Effects of external rhythmical cueing on gait in patients with Parkinson’s disease: a systematic review. Clin. Rehabil. 19, 695–713. 10.1191/0269215505cr906oa 16250189

[B33] London J. (2012). Hearing in Time. Oxford University Press

[B34] Madison G. (2006). Experiencing groove induced by music: consistency and phenomenology. Music Percept. 24, 201–208. 10.1525/mp.2006.24.2.201

[B35] Mazzoni P. Hristova A. Krakauer J. W. (2007). Why don’t we move faster? Parkinson’s disease, movement vigor, and implicit motivation. J. Neurosci. 27, 7105–7116. 10.1523/JNEUROSCI.0264-07.2007 17611263 PMC6794577

[B36] McIntosh G. C. Brown S. H. Rice R. R. Thaut M. H. (1997). Rhythmic auditory-motor facilitation of gait patterns in patients with Parkinson’s disease. J. Neurol. Neurosurg. Psychiatry 62, 22–26. 10.1136/jnnp.62.1.22 9010395 PMC486690

[B37] McKinney M. F. Moelants D. (2006). Ambiguity in tempo perception: what draws listeners to different metrical levels? Music Percep. Interdiscip. J. 24, 155–166. 10.1525/mp.2006.24.2.155

[B38] Meyer L. Cooper G. (1960). The Rhythmic Structure of Music. Chicago, IL: University of Chi-cago Press

[B39] Morris M. E. Iansek R. Matyas T. A. Summers J. J. (1994a). Ability to modulate walking cadence remains intact in Parkinson’s disease. J. Neurol. Neurosurg. Psychiatry 57, 1532–1534. 10.1136/jnnp.57.12.1532 7798986 PMC1073238

[B40] Morris M. E. Iansek R. Matyas T. A. Summers J. J. (1994b). The pathogenesis of gait hypokinesia in Parkinson’s disease. Brain 117, 1169–1181. 10.1093/brain/117.5.1169 7953597

[B41] Morris M. E. Iansek R. Matyas T. A. Summers J. J. (1996). Stride length regulation in Parkinson’s disease: normalization strategies and underlying mechanisms. Brain 119, 551–568. 10.1093/brain/119.2.551 8800948

[B42] Müllensiefen D. Gingras B. Stewart L. Musil J. (2012). The Goldsmiths Musical Sophistication Index (Gold-MSI): Technical Report and Documentation v1.0. London: Goldsmiths, University of London

[B43] Naugle K. M. Hass C. J. Joyner J. Coombes S. A. Janelle C. M. (2011). Emotional state affects the initiation of forward gait. Emotion 11, 267–277. 10.1037/a0022577 21500896

[B44] Nieuwboer A. Kwakkel G. Rochester L. Jones D. Van Wegen E. Willems A. M. (2007). Cueing training in the home improves gait-related mobility in Parkinson’s disease: the RESCUE trial. J. Neurol. Neurosurg. Psychiatry 78, 134–140. 10.1136/jnnp.200X.097923 17229744 PMC2077658

[B45] Nombela C. Hughes L. E. Owen A. M. Grahn J. A. (2013). Into the groove: can rhythm influence Parkinson’s disease? Neurosci. Biobehav. Rev. 37, 2564–2570. 10.1016/j.neubiorev.2013.08.003 24012774

[B46] Petrides M. Pandya D. N. (2006). Efferent association pathways originating in the caudal prefrontal cortex in the macaque monkey. J. Comp. Neurol. 498, 227–251. 10.1002/cne.21048 16856142

[B47] Phillips-Silver J. Toiviainen P. Gosselin N. Piche O. Nozaradan S. Palmer C. (2011). Born to dance but beat deaf: a new form of congenital amusia. Neuropsychologia 49, 961–969. 10.1016/j.neuropsychologia.2011.02.002 21316375

[B48] Rochester L. Baker K. Nieuwboer A. Burn D. (2011). Targeting dopa-sensitive and dopa-resistant gait dysfunction in Parkinson’s disease: selective responses to internal and external cues. Mov. Disord. 26, 430–435. 10.1002/mds.23450 21462258

[B49] Rochester L. Hetherington V. Jones D. Nieuwboer A. Willems A. M. Kwakkel G. (2005). The effect of external rhythmic cues (auditory and visual) on walking during a functional task in homes of people with Parkinson’s disease. Arch. Phys. Med. Rehabil. 86, 999–1006. 10.1016/j.apmr.2004.10.040 15895348

[B50] Roerdink M. Bank P. J. M. Peper C. L. E. Beek P. J. (2011). Walking to the beat of different drums: practical implications for the use of acoustic rhythms in gait rehabilitation. Gait Posture 33, 690–694. 10.1016/j.gaitpost.2011.03.001 21454077

[B51] Rossignol S. Jones G. M. (1976). Audio-spinal influence in man studied by the H-reflex and its possible role on rhythmic movements synchronized to sound. Electroencephalogr. Clin. Neurophysiol. 41, 83–92. 10.1016/0013-4694(76)90217-0 58771

[B52] Rubinstein T. C. Giladi N. Hausdorff J. M. (2002). The power of cueing to circumvent dopamine deficits: a review of physical therapy treatment of gait disturbances in Parkinson’s disease. Mov. Disord. 17, 1148–1160. 10.1002/mds.10259 12465051

[B53] Schwartze M. Keller P. E. Patel A. D. Kotz S. A. (2011). The impact of basal ganglia lesions on sensorimotor synchronization, spontaneous motor tempo, and the detection of tempo changes. Behav. Brain Res. 216, 685–691. 10.1016/j.bbr.2010.09.015 20883725

[B54] Sowinski J. Dalla Bella S. (2013). Poor synchronization to the beat may result from deficient auditory-motor mapping. Neuropsychologia 51, 1952–1963. 10.1016/j.neuropsychologia.2013.06.027 23838002

[B55] Spaulding S. J. Barber B. Colby M. Cormack B. Mick T. Jenkins M. E. (2012). Cueing and gait improvement among people with Parkinson’s disease: a meta-analysis. Arch. Phys. Med. Rehabil. 94, 562–570. 10.1016/j.apmr.2012.10.026 23127307

[B56] Stupacher J. Hove M. J. Novembre G. Schutz-Bosbach S. Keller P. E. (2013). Musical groove modulates motor cortex excitability: a TMS investigation. Brain Cogn. 82, 127–136. 10.1016/j.bandc.2013.03.003 23660433

[B57] Styns F. Van Noorden L. Moelants D. Leman M. (2007). Walking on music. Hum. Mov. Sci. 26, 769–785. 10.1016/j.humov.2007.07.007 17910985

[B58] Thaut M. H. Mcintosh G. C. Rice R. R. Miller R. A. Rathbun J. Brault J. M. (1996). Rhythmic auditory stimulation in gait training for Parkinson’s disease patients. Mov. Disord. 11, 193–200. 10.1002/mds.870110213 8684391

[B59] Wittwer J. E. Webster K. E. Hill K. (2013). Music and metronome cues produce different effects on gait spatiotemporal measures but not gait variability in healthy older adults. Gait Posture. 37, 219–222. 10.1016/j.gaitpost.2012.07.006 22871238

[B60] Yogev G. Giladi N. Peretz C. Springer S. Simon E. S. Hausdorff J. M. (2005). Dual tasking, gait rhythmicity, and Parkinson’s disease: which aspects of gait are attention demanding? Eur. J. Neurosci. 22, 1248–1256. 10.1111/j.1460-9568.2005.04298.x 16176368

