# Supplementary material for: Individual Differences in Beat Perception Affect Gait Responses to Low- and High-Groove Music
Source: Front Hum Neurosci. 2014 Oct 22;8:811. doi: 10.3389/fnhum.2014.00811 (PMC4205839; doi:10.3389/fnhum.2014.00811)
Supplement: Supplementary file 1 [file Table_1.DOCX]

Stimuli Selection:

A database of 49 song clips was compiled by study author 2. Eleven lab members rated these clips in terms of groove, familiarity and enjoyment. Stimuli rated as greater than 5 on familiarity was discarded. A set of 20 clips with ten lowest and ten highest groove ratings were selected. The set of 20 clips are listed below. Tempos for these 20 clips were estimated by having participants manually tap to the beat on a USB-connected keyboard. For participant ratings, intraclass correlation coefficient was high for groove (r=.944), and moderate for familiarity (r=.857) and enjoyment (r =.758)

| **Song Title** | **Artist** | **Album** | **Estimated Tempo** |
| --- | --- | --- | --- |
| Ritmo Caliente | Eddie Palmieri | Sugar Daddy | 96 |
| Rodeo: Hoe-down | Michael Tilson Thomas | Copland: The populist | 130 |
| Remember | ATB (André Tanneberger) | Dedicated | 127 |
| Bryter Layter | Nick Drake | Bryter Layter | 122 |
| Buckethead | Jordan | Jordan | 116 |
| Clubbed to Death | Rob Dougan | Cool Chillout | 101 |
| Conmigo | Eddie Palmieri | Sugar Daddy | 96 |
| Jumpin at the woodside | The Count Basie Orchestra | 1937-1943 | 121 |
| Drifting | Andy McKee | Art of Motion | 127 |
| Druid Fluid | Yo-Yo Ma, Edgar Meyer, Mark O’Connor | Appalachia Waltz | 117 |
| Extreme Ways | Moby | 18 | 101 |
| Falling Blue - Beautiful R&B Instrumental | Farhan Khan | N/A (single) | 92 |
| Farewell | Apocalyptica | Apocalyptica | 98 |
| The Flik Machine | Randy Newman | A Bug's Life | 124 |
| Halo | Michael Salvatori | Halo: Original Soundtrack | 126 |
| Unstoppable | E.S. Posthumus | Makara | 110 |
| Hideaway | John Mayall & the Blues Breakers, Eric Clapton | Bluesbreakers | 149 |
| Mombasa | Hans Zimmer | Inception | 110 |
| Fortuna | Kaki King | Everybody Loves You. | 106 |
| Blues For Two (Instrumental) | Johnny Cash cover by Luther Perkins | 100 American Country | 96 |
| Primavera | Ludovico Einaudi | Divernire | 138 |
| Savor | Santana | Santana | 135 |
| Treat | Santana | Santana | 141 |
| Ray Dawn Balloon | Trey Anastasio | Trey Anastasio | 110 |
| bgmusic | Catherine Michael | N/A (single) | 125 |
| FL Studio - Acoustic Guitar, Violin | Farhan Khan | N/A (single) | 96 |
| Love Instrumental -- Piano Beat | GNG Instrumentals | N/A (single) | 126 |

Supplementary Materials Table 1.Exploratory analyses of asynchronies (i.e., the phase difference between the beat time and the step time). Similar to previous studies ([Fujii and Schlaug, 2013](#_ENREF_17); [Sowinski and Dalla Bella, 2013](#_ENREF_55); [Launay et al., 2014](#_ENREF_31)), asynchronies were converted into phase angles by first obtaining the signed difference between the beat time and the step time, then normalized to the nearest interstep interval, and finally transforming into a circular scale by multiplying by 360°. Phase angle values between 180-360° indicate that the step preceded the beat, values between 0-180° indicate that the step came after the beat. Phase matching variability was estimated with the r-vector length, where the longer the r-vector, the more consistent was phase-matching performance. Group mean phase angle and variability were averaged from the most consistent trial (trials with longer r-vector) for each of the different cue types at preferred tempo from each participant. These analyses were not run for the 22.5% faster condition due to excessive variability (r-vectors shorter than 0.45 in majority of trials).

|  | Low Groove | High Groove | Metronome |
| --- | --- | --- | --- |
| Group mean phase angle (n=26) (synchronization accuracy) | M= 0.31°  95% CI: [52.4°, 82.4°] | M= 356.85°  95% CI: [43.5°, 281.8°] | M=18.58°  95% CI: [39.5°, 359.4°] |
| Length of R-vector (n=26) (synchronization variability) | 0.63 | 0.75 | 0.80 |
